# Improvement in Luminous Efficacy and Thermal Performance Using Quantum Dots Spherical Shell for White Light Emitting Diodes

**DOI:** 10.3390/nano8080618

**Published:** 2018-08-15

**Authors:** Songmao Chen, Caiman Yan, Yong Tang, Jiasheng Li, Xinrui Ding, Longshi Rao, Zongtao Li

**Affiliations:** 1 Key Laboratory of Surface Functional Structure Manufacturing of Guangdong High Education Institutes, South China University of Technology, Guangzhou 510641, China; smchen@scut.edu.cn (S.C.); chamenyan@163.com (C.Y.); ytang@scut.edu.cn (Y.T.); jiasli@foxmail.com (J.L.); ding.xinrui@mail.scut.edu.cn (X.D.); memerls@mail.scut.edu.cn (L.R.); 2 Foshan Nationstar Optoelectronics Company Ltd., Foshan 528000, China

**Keywords:** chip-on-board, quantum dots, spherical shell structure, white light-emitting diode

## Abstract

White light-emitting diodes (WLEDs) based on quantum dots (QDs) are gaining increasing attention due to their excellent color quality. QDs films with planar structure are universally applied in WLEDs for color conversion, while they still face great challenges in high light extraction and thermal stability. In this study, a QDs film with a spherical shell structure was proposed to improve the optical and thermal performance for WLEDs. Compared with the conventional planar structure, the luminous efficacy of the QDs spherical shell structure is improved by 12.9% due to the reduced total reflection effect, and the angular-dependent correlated color temperature deviation is decreased from 2642 to 283 K. Moreover, the highest temperature of the WLED using a QDs spherical shell is 4.8 °C lower than that of the conventional WLED with a planar structure, which is mainly attributed to larger heat dissipation area and separated heat source. Consequently, this QDs spherical shell structure demonstrates superior performance of QDs films for WLEDs applications.

## 1. Introduction

White light-emitting diodes (WLEDs) have attracted significant attention as superior light sources due to their low cost, high luminous flux, low energy consumption, and ecological environment protection [1]. WLEDs play an increasingly important role in our daily life by being applied in many fields, such as general lighting, medical, and lifestyle products [2]. The luminous efficacy, color temperature, color gamut, and thermal management are important parameters for evaluating WLEDs [3,4]. There have been many techniques reported for improving their luminous efficacy, such as using a distributed Bragg reflector [5], adding TiO_2_ or SiO_2_ into the encapsulation layer [6,7], manufacturing a textured phosphor structure by the imprinting technique [8], and so on [9]. A higher luminous efficacy means a higher light extraction from the LED device, which is beneficial for their applicability.

Conventionally, a WLED is obtained by combining yellow phosphor with a blue light-emitting diode, such as a Yttrium Aluminum Garnet (YAG) phosphor [10]. However, this traditional phosphor, possessing a narrow excitation width and broad full width at half maximum (FWHM) of about 100 nm [11], is suboptimal for WLEDs design. As quantum dots (QDs) technology experiences rapid development [12,13,14], QDs, such as, CdSe/ZnS, exhibit a narrow FWHM (~30 nm), high absorption coefficient, tunable band gap, wide color gamut, and high quantum yield of up to 80%, which renders them more suitable for LED devices [15]. Replacing the phosphor powder with QDs as the fluorescence material for LED devices has recently become a topic of interest [16,17]. After combination with blue or ultraviolet (UV) LEDs, the QDs have been packaged in silicone as a planar film for LED applications, creating a white light with the desired color temperature [18,19]. A color gamut performance greater than 100% of the National Television Standards Committee (NTSC) TV color standard (1953) has been achieved using narrow-line width red and green QDs [20,21,22]. However, like the phosphor WLED, the QDs package used for WLEDs fabrication still requires improvements for achieving higher luminous efficacy [23].

Thermal management has a strong effect on the performance of WLEDs, especially QDs-WLEDs. Ju Yeon Woo et al. [24] demonstrated that the photon energy absorbed by the QDs is lost as heat through electron-phonon scattering during emission. In addition, the heat generation of the QDs is significantly higher than that of phosphor of the same weight [24]. QDs are sensitive to heat, as heat accumulation causes an increase in temperature. Excessive temperature lowers the light conversion efficacy of QDs. It is necessary to reduce the effect of temperature on the QDs to increase the light conversion efficacy and achieve a high lumen WLED [25]. Kuo-Ju Chen et al. indicated that QDs have significant application potential in LEDs if their thermal environment is improved [26]. However, previous studies on QD-based WLEDs were mainly focused on the planar QD film without considering its geometry [16,18,27,28], which suppresses their potential improvement in the optical and thermal performance.

In this study, we propose a spherical-shell structure to improve the thermal management and luminous efficacy for QDs-WLEDs. By using different molding dies, CdSe/ZnS QDs mixed with polydimethylsiloxane are shaped as a spherical shell film or a conventional planar film. After combining with a blue chips-on-board (COB), two different WLEDs are fabricated. Their optical and thermal properties are studied under similar total correlated color temperature (CCT).

## 2. Methods

**Figure 1:**
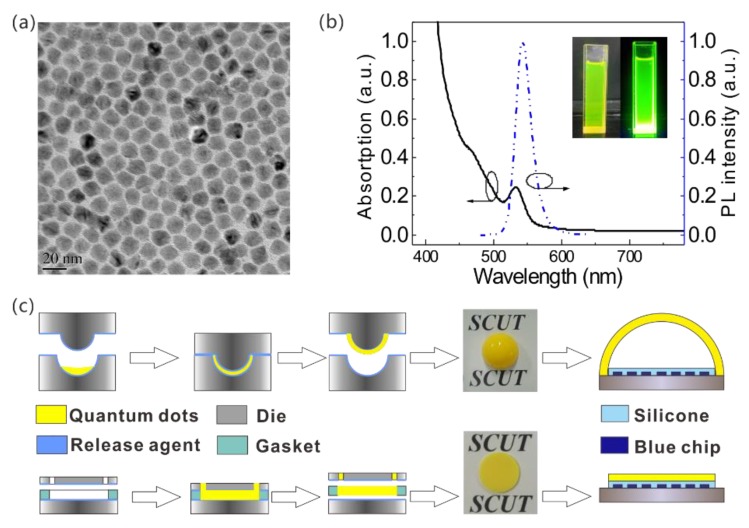
(**a**) Transmission electron microscopy (TEM) image of the CdSe/ZnS quantum dots; (**b**) Ultraviolet (UV)-visible absorption and PL spectrum of green-emitting CdSe/ZnS quantum dots; (**c**) Fabrication processes of WLEDs with spherical-shell and planar quantum-dot film.

The CdSe/ZnS quantum dots used in the present work were bought from Beijing BEIDA JUBANG SCIENCE & TECHNOLOGY Co., Ltd. (Beijing, China), with an experimental photoluminescence quantum yield (PLQY) of 82%. The transmission electron microscopy (TEM) image of the CdSe/ZnS QDs is shown in Figure 1a. The prepared QDs exhibit good monodispersion with an average size of 14 nm. The UV-visible absorption and photoluminescence (PL) emission spectra of the CdSe/ZnS QDs dispersed in chloroform are shown in Figure 1b. The PL emission peak is located at 542 nm and possesses a 10 nm red-shift relative to the absorption peak located at 532 nm, which is attributed to Stokes-shift. Its FWHM is 27 nm under an excitation wavelength of 365 nm. The absorption spectrum reveals that light in the 380–532 nm wavelength range, including blue LED emission, can be absorbed efficiently by CdSe/ZnS QDs.

**Figure 2:**
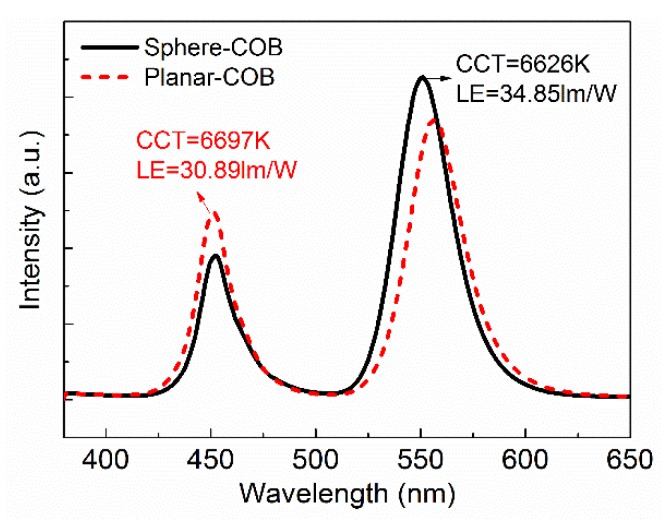
Emission spectra of sphere-(chips-on-board) COB and planar-COB.

Using different molding dies, the spherical-shell QDs film or the conventional planar QDs film can be prepared. As shown in Figure 2, a release agent was placed on the inner surface of the dies first. Subsequently, for the spherical-shell QDs film, the CdSe/ZnS QDs mixed with polydimethylsiloxane (PDMS) were injected into the die cavity before the upper die closed the die cavity. For the conventional planar quantum film, a gasket was placed between the dies to form the cavity and the same mixture was injected through the upper hole. After annealing the die cavities for 1 h at 110 °C, the finished spherical-shell and planar QDs films could be released. The prepared QDs films were assembled on the same chips-on-board (COB) light source, which was formed by 42 pieces of square blue LED chips bonded in a 6 × 7 array with an emission wavelength of 450 nm, packaged in silicone. After the assembly, the COBs with the prepared film were installed on a heat sink for stabilizing the junction temperature.

For the ultraviolet visible spectroscopy, firstly, the QDs were dissolved in chloroform solution. Secondly, scanning the pure chloroform as the baseline, the ultraviolet visible absorption spectra of QDs was measured by using a UV–vis spectrometer (UV–2600, Shimadzu, Kyoto, Japan). For the photoluminescence spectroscopy, using the dispersed QDs solution, the photoluminescence spectra of the QDs was recorded by a fluorescence spectrophotometer (RF-6000, Shimadzu, Kyoto, Japan) with a Xe lamp under an excitation located at 365 nm. For transmission electron microscopy, the QDs samples were prepared by dropping a diluted dispersion of QDs-chloroform solution on a carbon-coated copper grid, followed by a solvent evaporation process under heated condition at 80 °C. Transmission electron microscope (TEM) images were taken by a transmission electron microscope (TEM, JEM-2100F, JEOL, Tokyo, Japan) operated at an accelerating voltage of 200 kV. The emission spectra of the COB devices were measured by an integrating sphere system. The angular-dependent correlated color temperature, as well as the light intensity were tested by a fluorescence spectroscopy photon detector. To expand the thermal tests, the original COB, Sphere-COB and Planar-COB were fixed at the same place away from the infrared thermal imaging instrument (A655SC, FLIR SYSTEMSAB, Seattle, WA, USA). The camera of the instrument was vertically focusing on those devices. Every test was carried out under a constant current drive of 100 mA supported by a power supply (Keithley 2425, Beaverton, OR, USA).

## 3. Results and Discussion

Table 1 shows a comparison between the WLEDs with the conventional planar film referred to as planar-COB and spherical shell film structure referred to as sphere-COB. The total correlated color temperature (CCT) of the sphere-COB and the planar-COB is 6626 K and 6697 K, respectively. As is shown in Figure 2, the blue and green peaks are derived from the chips and QDs, respectively. With a similar CCT, the luminous efficacy (LE) of the sphere-COB is 34.85 lm/W, about 12.9% higher than that of the planar-COB (30.89 lm/W). Under the same amount blue-light excitation, the sphere-COB shows lower blue-light and higher green-light. This improvement in the sphere-COB is primarily attributed to the higher light conversion efficacy from blue to green light, which matches their color coordinates: (0.299, 0.394) for the planar-COB and (0.290, 0.461) for the sphere-COB. In addition, the total radiation power of the device is also improved from 0.122 W of the planar-COB to 0.125 W of the sphere-COB, indicating that the energy loss of the sphere-shell film is lower. This also contributes to the increased luminous efficacy of the sphere-COB.

**Figure 3:**
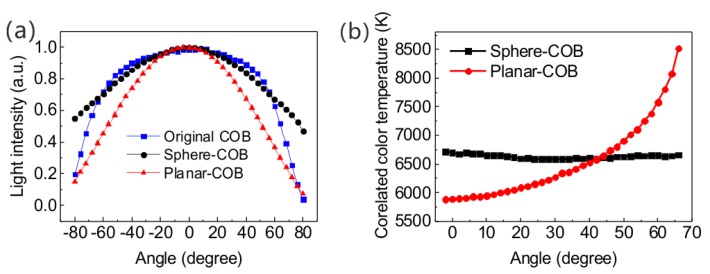
(**a**) Normalized light intensity distribution of the three COBs; (**b**) The angular-dependent CCT of the sphere-COB and planar-COB.

Figure 3a shows the angular-dependent normalized light intensity distribution of the three COBs. The original COB represents the original blue-chip COB without any QDs film. At the edge angle of 80°, the relative intensities of the original COB, planar-COB, and sphere-COB are 3.8%, 7%, and 47%, respectively. This means that the sphere-shell film can emit more green light at the edge angle. The device with the planar QD film focuses most of its emitted light in the center. On the contrary, the sphere-COB emits more lights at the edges. In other words, the spatial light distribution narrows with the planar-COB and broadens with the sphere-COB, relative to the original COB. The beam angle is defined as the angle between the light intensity values equal to half of the maximum intensity. Hence, the beam angles of the original COB, sphere-COB, and planar-COB are about 140°, 160°, and 115° respectively, indicating that the sphere-COB can broaden the beam angle by 20° relative to the original COB. The angular-dependent CCTs of the sphere-COB and the planar-COB are shown in Figure 3b. It can be observed that the CCT deviation drops significantly from 2642 K (planar-COB) to 283 K (sphere-COB). The planar-COB CCT achieves its lowest value at the center and gradually increases toward the edge while the sphere-COB exhibits a fairly uniform CCT. This experimental result reveals that the sphere-COB possesses a superior color temperature uniformity and indicates that the broader beam angle is beneficial for forming a uniform CCT.

**Figure 4:**
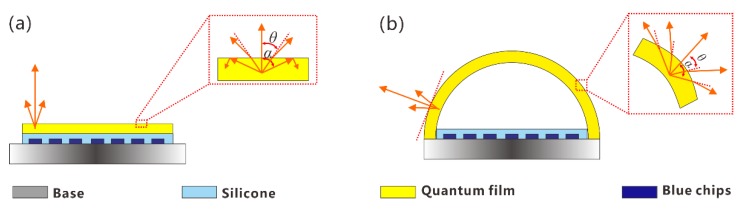
The optical model of planar-COB (**a**) and sphere-COB (**b**), respectively. The enlarged rectangle represents the unit luminescence mechanism.

**Figure 5:**
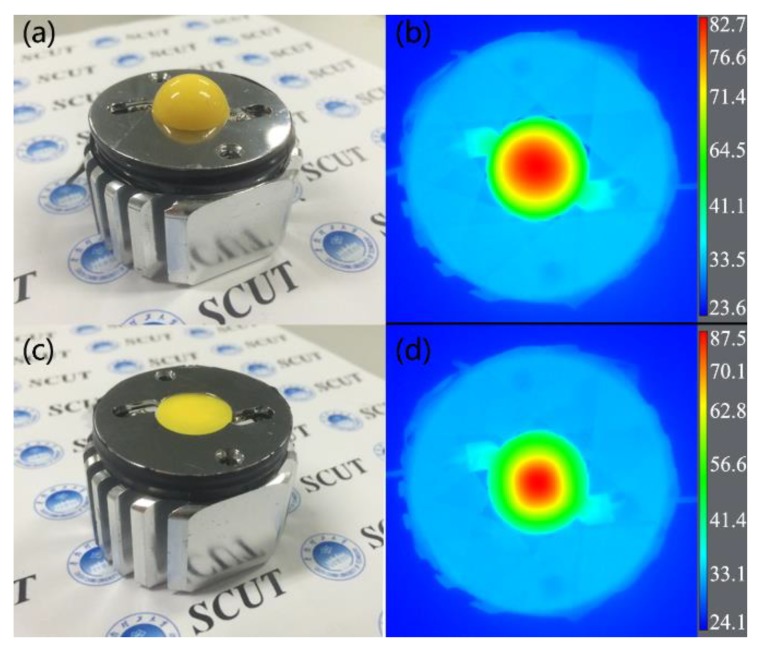
(**a**) Photograph and (**b**) infrared thermal imaging of the sphere-COB; (**c**) photograph and (**d**) infrared thermal imaging of the planar-COB.

To understand the underlaying optical mechanisms, a model of the planar-COB and sphere-COB is shown in Figure 4. After being combined in the planar or sphere-shell films, the QDs turn a portion of the blue light into green light resulting in the emission of white light with different light distributions [29]. For the planar-COB, as shown in Figure 4a, the incident angle between the green light and the plane increases gradually from the center towards both sides. The critical angle can be calculated by using the total reflection formula:

(1) $$\alpha =\arcsin(n_{2}/n_{1})$$

where *n_1_* and *n_2_* represent the refractive index of air and PDMS, respectively. The critical angle α of the considered film is about 45.4°. When the incident angle is over this critical angle, total reflection occurs, reducing the light output. Especially at the edges, this phenomenon is considerable, resulting in the light intensity approaching zero. For the sphere-COB, the enlarged rectangle in Figure 5b shows that the green light has a lower reflection compared with the planar-COB due to the curved interface reducing the angle between the incident light and the plane. Hence, more green light can be extracted from directions above the critical angle in the sphere-COB. The reduced total reflection loss in the sphere-COB explains the improved luminous efficacy.

The strongest light output of a film is along the line normal to the surface. For the planar-COB, the green light is more likely to be emitted in the vertical direction, as it coincides with the direction normal to the film. Hence, the light is focused at the center, resulting in a maximum emission, which decreases towards the edges. In contrast, the normal line of the sphere-COB is distributed along the curved surface so that the green light can emit along the sphere curve, which increases the light intensity of the large angle. This is consistent with the experimental data showing that the sphere-COB can emit more light at the edge, as shown in Figure 3a. This difference explains the increased beam angles of the sphere-COB. As a result, by taking advantage of the curved shape of the sphere-COB, the CCT uniformity can be significantly improved without additional scattering elements [30].

To investigate the thermal performance of the two considered structures, infrared thermal imaging of the sphere-COB and planar-COB in stable working conditions was shown in Figure 5. The highest temperature of each device is located at its center. The highest temperature of the sphere-COB is 82.7 °C, which is 4.8 °C lower than the 87.5 °C achieved by the planar-COB. The heat concentrated in the yellow-red circle represents the majority of the heat generated by the devices and the yellow-red circular area of the sphere-COB is about 1.3 times larger than that of the planar-COB. This indicates that the sphere-COB has a larger heat dissipation area. The larger heat dissipation area of sphere-COB contributes to the improved thermal management of the device, resulting in a decreased highest temperature.

**Figure 6:**
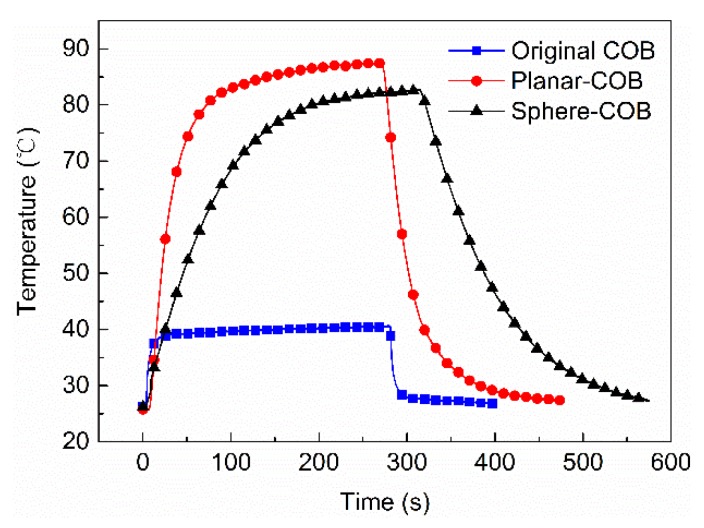
Temperature curve of the original COB, sphere-COB, and planar-COB.

Figure 6 shows the highest temperature located in the center region of three different COBs as a function of time to expand the thermal test. After switching the devices on at 0 s, the planar-COB achieves 80 °C from room temperature in only 72 s, while the sphere-COB requires 195 s. Under stable working conditions, the highest temperature of the original COB, sphere-COB, and planar-COB is 40.7 °C, 82.7 °C and 87.5 °C, respectively. The original COB is composed of the blue chips and the silicone without any films. As a result, it can only emit the blue light, which means no short-wavelength light turns into green light unlike the sphere-COB or the planar-COB, which explains its lowest temperature at stable state. The cooling process exhibits a similar trend to the heating process. The average cooling speed from the highest temperature to 30 °C of the original COB, planar-COB, and sphere-COB is about 0.89, 0.78, and 0.41 °C/s, respectively. Not only does the planar-COB have a higher temperature than the sphere-COB in its stable state, the planar-COB is also subject to more rapid heating and cooling, similarly to the original COB.

There are two primary reasons for this superior thermal performance of the sphere-COB. The first is its larger heat dissipation area, as discussed above. A larger area is beneficial for the heat dissipation. The other important reason is that the heat source is separated from the spherical-shell film in the sphere-COB structure. There is no direct contact between the spherical-shell film and heat source. In contrast, the planar film is in direct contact with the heat source, resulting in a rapid temperature increase. In addition, total reflection causes the reflected light, which would be absorbed by the QDs [31], to increase heat inside the structure. Therefore, the lower reflection in the sphere-COB aids the reduction of heat generation. The resulting improved thermal management of the sphere-COB is conducive to improve the QDs conversion efficacy and subsequently the luminous efficacy of the WLED. As QDs are quite sensitive to the heat [32], this improvement has significance on the QDs applicability, on one hand, total electrical energy is mainly transforming into light and heat, more light could be given out after heat dissipation is improved. On the other hand, a lower temperature is beneficial for the service life of the device [33].

## 4. Conclusions

In this study, we proposed a spherical-shell QDs film structure for COB-WLEDs. Compared with the conventional planar QDs film structure under similar CCT, the luminous efficacy was improved by 12.9%, because the reduced total reflection in the spherical-shell film causes a higher green light extraction. In addition, the spherical shell structure increased the beam angle by 20°, resulting in a CCT deviation of only 283 K. The sphere-COB exhibits excellent heat dissipation and achieves a maximum temperature 4.8 °C lower than that of the planar-COB, this is because that the spherical-shell film has a large area for convention heat transfer and been isolated from the heat source of LED chips by air. Hence, the novel structure can attain enhanced luminous efficacy and outstanding heat dissipation capabilities for WLEDs, which can provide a new prospective to improve WLEDs by optimizing the geometries of QD films. 

## Figures and Tables

**Table 1:** Comparison of conventional planar-COB and sphere-COB.

Sample	Planar-COB	Sphere-COB
Luminous efficacy (lm/W)	30.89	34.85
Improved LE (%)	Reference	12.9
Chromaticity coordinate (X)	0.299	0.290
Chromaticity coordinate (Y)	0.394	0.461
CCT (K)	6626	6697
ΔCCT (K)	2642	283
Beam angle (°)	115	160
Highest temperature (°C)	87.5	82.7
Decreased temperature (°C)	Reference	4.8
